# Immune System Modulation and Viral Persistence in Bats: Understanding Viral Spillover

**DOI:** 10.3390/v11020192

**Published:** 2019-02-23

**Authors:** Sonu Subudhi, Noreen Rapin, Vikram Misra

**Affiliations:** Department of Microbiology, Western College of Veterinary Medicine, University of Saskatchewan, Saskatoon, SK S7N 5B4, Canada; noreen.rapin@usask.ca (N.R.); vikram.misra@usask.ca (V.M.)

**Keywords:** Immune response, flight, immune tolerance, viral persistence, spillover, bats, viruses

## Abstract

Bats harbor a myriad of viruses and some of these viruses may have spilled over to other species including humans. Spillover events are rare and several factors must align to create the “perfect storm” that would ultimately lead to a spillover. One of these factors is the increased shedding of virus by bats. Several studies have indicated that bats have unique defense mechanisms that allow them to be persistently or latently infected with viruses. Factors leading to an increase in the viral load of persistently infected bats would facilitate shedding of virus. This article reviews the unique nature of bat immune defenses that regulate virus replication and the various molecular mechanisms that play a role in altering the balanced bat–virus relationship.

## 1. Introduction

Over the past few years, the interest in bat research has increased because of the range of viruses that they harbor and the potential of spillover to humans and other vertebrates [1]. In some instances, there is direct evidence that viruses have spilled over from bats to other vertebrates. For example, Nipah virus spilled over from bats to humans and pigs leading to outbreaks in Southeast Asia and Hendra virus continues to spillover into horses and humans in Australia [2,3,4,5,6]. In other instances, the evidence of virus spillover from bats is circumstantial. For example, in case of ebolaviruses, serological evidence and the detection of viruses related to the ebolaviruses in bats [7,8] suggests that they are reservoirs of the virus. Recent discoveries of filoviruses in bats of China and Sierra Leone have further bolstered this claim [9,10]. However, viruses linked to specific Ebola virus disease (EVD) epidemics have not yet been detected in bats. Although bats harbor many diverse viruses, their spillover to other animals is extremely rare [11]. One of the reasons is that for a spillover event to take place several factors such as pathogen shedding, environmental conditions, pathogen persistence in environment, and recipient host susceptibility, should all be conducive to transmission. This would result in the “perfect storm” required for spillover. A review by Plowright et al. [12] describes the factors that could lead to such a perfect storm. Here, we focus on one of the initial events affecting this spillover, i.e., an increase in pathogen shedding.

Pathogen shedding by bats is a complex phenomenon regulated by host antiviral defenses and viral factors. A host’s defenses attempt to control virus infection but viruses have evolved mechanisms to counteract them [13]. When the host defenses are suppressed, or if the virus is able to circumvent the host immune system, virus replication is enhanced, leading to an increase in virus shedding. The route of shedding is highly dependent on the tissue in which the virus replication occurs. For example, increased replication in the kidney would lead the bat to shed the virus in urine, whereas intestinal replication would result in dissemination through feces.

Therefore, it becomes important to study what factors might be responsible for decreasing the host defense or factors that allow the virus to circumvent host immune response. But before exploring factors affecting virus–host interactions, it is imperative to understand the viruses present in bats and the bat immune defenses. In 2013–2014, bat immunology was at a nascent stage and Schountz and Baker et al. [14,15] had summarized the preliminary knowledge about it in their reviews. In 2017, Schountz et al. [16] again reviewed bat immunology but the article also laid out gaps in our understanding of host pathogen interactions in bats. Here, we include more recent advances in characterizing the bat immune system including a possible link between the evolution of flight in bats and viral persistence. Following that section, we discuss the factors identified so far that upset the balanced bat–virus relationship, potentially leading to the spillover of viruses.

One must be cautious when interpreting the literature on bat defenses. The order Chiroptera contains more than 1200 species [17]. Due to this diversity, the information obtained from one species may not apply to others. For instance, in the black flying fox (*Pteropus alecto*), the interferon gene locus is contracted, whereas in the Egyptian fruit bat (*Rousettus aegyptiacus*), this locus is expanded [18,19]. Other examples are discussed later in this review.

## 2. Bats Have an Efficient and Varied Antiviral Response

It has been observed that viruses which severely affect other mammals, including humans, are apparently nonpathogenic for bats [20]. This adaptability of bats to harbor many viruses without showing overt pathology suggests that bats have evolved immune mechanisms that allow for benign virus–host relationships.

The immune response has two primary components, innate and adaptive. The host initially responds to infection by activating innate mechanisms. Genes such as those for sensing and repairing DNA damage and the inflammatory process are under positive selection in black flying foxes and David’s myotis (*Myotis davidii*) [21]. For instance, there are mutations in the coding sequence of p53 functional domains that are unique to these bats. Interferons are the primary innate effector molecules that control viral replication. Several types of interferon have been identified in bats, especially Type I and Type III. Type I interferon is induced after virus infection in Egyptian fruit bats whereas Type III interferon is induced similarly in pteropid bats [22,23]. It is interesting to note that interferon genes in black flying foxes have contracted in terms of diversity. The number of variants of interferon present in these bats is lower compared to those in the gene loci of ten other vertebrate species. Despite this decrease in interferon gene diversity, black flying foxes express these variants at higher basal levels than other mammalian species. This suggests that the interferon and interferon stimulated genes are constitutively expressed in these bats [18]. In contrast, a recent study of the genome of Egyptian fruit bats shows expanded diversity of Type I interferon genes. This, as well as expansion of various other immune genes, suggests novel modes of antiviral defense in bats of various species [24]. Novel modes of antiviral defenses could exist in other species of bats and future studies would help in revealing them.

Cruz-Rivera et al. [25] showed that in cultured cells of black flying foxes, interferon-stimulated genes (ISGs) were expressed at higher levels than their human counterparts. They also demonstrated that an antiviral effector 2-5A-dependent endoribonuclease (RNase-L) is a unique downstream ISG gene, which is stimulated directly by interferon in bat cells [25]. The activated RNase-L can then cleave viral mRNA. In contrast, in human cells, RNase-L is not directly stimulated by interferon but via an intermediate molecule, 2′,5′-oligoadenylate (2-5A) synthetase [26]. Similarly, direct activation of RNase-L was also observed in in vivo experiments with Jamaican fruit bats (*Artibeus jamaicensis*) [27]. Inactivation of the RNase-L gene renders bat cells more susceptible to virus infection and direct induction of RNase-L may provide bats with an additional layer of antiviral defense [25].

These studies suggest that in bats of several species, higher level of interferon and ISGs are always present in their cells, which makes them better prepared to control viruses. Overall, bats seem to possess either an “always ON” interferon strategy plus or a better antiviral ISG defense strategy.

## 3. Bats Suppress the Pathological Effects of Excessive Virus-Induced Inflammation

While bats are well prepared to control viral infections, they also have mechanisms to avoid over-induction of inflammatory genes. Such excessive inflammation is detrimental in vertebrates of other species, such as humans, and is linked to pathology [28]. So, it is beneficial for the bats to have evolved mechanisms that would allow them to control excessive inflammation. Cell culture experiments have shown that cells of several species of bats have an inhibitor molecule (cRel) binding site in the promoter region of tumor necrosis factor alpha (TNFα), a key inflammatory cytokine. In cells of big brown bats (*Eptesicus fuscus*), stimulated with poly I:C, a surrogate for double-stranded viral RNA, cRel actively suppresses TNFα expression [29]. Genome analysis of black flying foxes and David’s myotis have also demonstrated positive selection pressure on the cRel gene [21]. This suggests that many bats have a mechanism to suppress the expression of TNFα, thereby maintaining a balanced response to viral infection.

Several species of bats control inflammation by having a mutation at a highly conserved serine residue in one of the key adaptor molecules for sensing damaged DNA, i.e., stimulator of interferon genes (STING), which reduces its functionality. STING senses damaged DNA or dsDNA from viruses and induces an interferon response. The replacement of serine at position 358 by other amino acids makes bat STING less effective in activating interferons [30]. In addition to possessing a less effective STING, the pyrin and HIN domain (PHYIN) genes, which are involved in microbial DNA sensing and formation of inflammasome, are absent in bats [21,31]. These findings demonstrate some of the reasons for reduced inflammation in bats during viral infection. Mechanisms to modulate inflammation may have evolved to mitigate the detrimental effects of flight. Excessive exposure to cytosolic DNA in bat cells during flight might have posed a strong natural selection pressure to reduce the activation of bat DNA sensors.

### Unique Immune Features and Relationship with the Evolution of Flight

**Figure 1:**
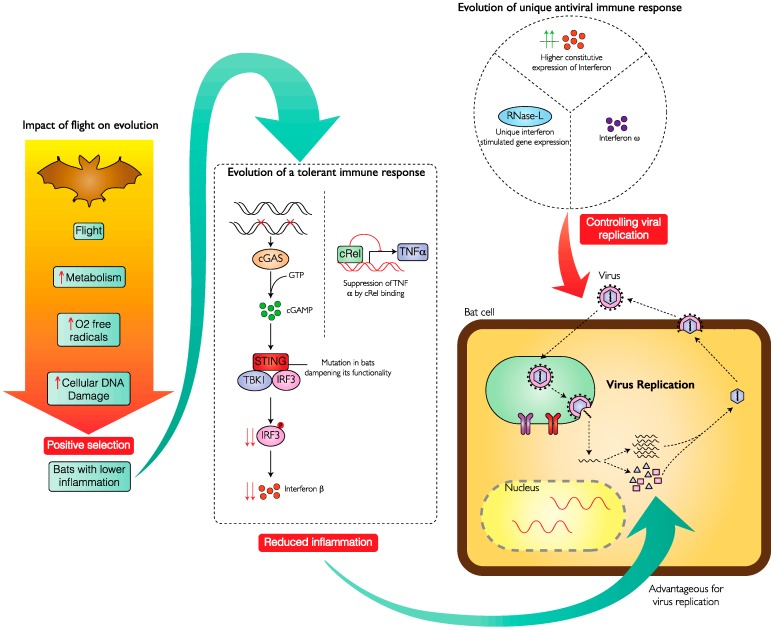
Evolution of tolerance to DNA damage and unique antiviral immune response in bats. Development of flight necessitated the evolution of bats with the ability to modulate the consequences of increased metabolic activity by suppressing inflammation (left). Inflammation was suppressed by dampening the activation of DNA sensors, such as STING, and reducing levels of inflammatory cytokines, such as TNFα (center). These traits were positively selected but a reduced inflammatory response made it advantageous for virus replication (lower right). Increased susceptibility of cells to virus replication was compensated by selection of more effective antiviral measures, such as higher constitutive expression of Interferons or unique ISG expressions (upper right). (Abbreviations used: cGAS—cyclic GMP-AMP synthase, GTP—Guanosine triphosphate, cGMP—cyclic guanosine monophosphate, STING—stimulator of interferon genes, TBK1—TANK binding kinase 1, IRF3—interferon regulatory transcription factor 3, cRel, TNFα—tumor necrosis factor α, RNase-L—ribonuclease L).

The unique features of bats, whereby they tolerate viral infections without excessive inflammation while suppressing viral replication, leads to the obvious question: What is so special about bats? The answer to this has been linked to the evolution of the ability to fly. The increased rate of metabolism accompanying flight would lead to higher levels of oxygen-free radicals [32,33]. This makes bats more prone to generating damaged DNA [34]. As mounting an immune response is energetically expensive [35] and would be detrimental, bats probably evolved mechanisms to suppress activation of immune response due to damaged DNA generated via flight, thereby leading to reduced inflammation. This would also explain why bats of certain species live longer than expected given their high metabolism and small size [36]. In bats the evolutionary suppression of inflammation and consequent susceptibility to virus infection is counteracted by constitutive expression of innate immune genes or novel genes to target viruses as described earlier. This model has been depicted in in Figure 1.

## 4. Viral Persistence in Bats

Over the past few years, numerous viruses have been detected in bats and these viruses seldom cause any overt disease (with the exception of Tacaribe virus and rabies virus) [1,16,37]. Detection of virus and absence of disease have led researchers to suggest that bats are likely the reservoirs of these viruses. Asymptomatic infections have been observed in bats for human pathogens such as henipaviruses (Nipah and Hendra viruses), coronaviruses (Middle Eastern Respiratory Syndrome coronavirus (MERS-CoV)), and filoviruses (Marburg virus and ebolaviruses) [38,39,40]. For a species to be a viral reservoir, the virus needs to persist in the population. Two probable ways in which this can happen are (1) virus infection and clearing from infected individuals is an ongoing process and introduction of naïve individuals maintains the virus in the population and (2) individuals infected with the virus are able to maintain the virus in the form of a persistent infection. Although either or both of these possibilities may influence the bat–virus relationship, there is considerable evidence for the maintenance of some viruses in bat populations by continued, low-level persistence.

A study done by Sohayati et al. [41], done on captive large flying foxes (also known as Malayan flying fox; *Pteropus vampyrus*), showed the possibility of recrudescence of Nipah virus (NiV). Regular sampling was done for over a period of one year to study the presence of virus and level of antibody against it. The authors discovered that one bat had a waning antibody titer with subsequent detection of virus in the urine. Following virus shedding, the antibody levels increased in this animal. As virus was undetectable in earlier samples of the same bat, the authors suggest that the virus may have persisted, probably in certain organs or cells, rendering it undetectable in blood, throat swab or urine. Within two weeks after NiV was isolated from the bat, two other male bats seroconverted and demonstrated an increase in antibody titers, suggesting that recrudescence led to horizontal transmission.

Other studies on Marburg virus transmission among Egyptian rosette bats (*R. aegyptiacus*) showed that bats naturally infected by other experimentally inoculated bats, seem to have a prolonged incubation period [42,43]. Furthermore, the infected bats remained viremic and shed infectious virus for up to three weeks, after which there was no detectable virus in blood, oral swabs and urine samples. Despite the lack of detectable virus, even four months after initial infection, experimentally inoculated bats were able to transmit the virus to other contact bats [43]. One of the ways in which this can be explained is that Marburg virus persisted in the bats (probably in the spleen [42]), and a decrease in antibody levels led to increase in viral load which could then be shed to infect other bats/animals.

A bat coronavirus was also shown to persistently infect North American little brown bats (*Myotis lucifugus*) [44]. Little brown bats in captivity were able to harbor the coronavirus in their intestines and lungs during hibernation for a period of four months. In addition, there was no significant pathology seen in the bat tissues.

To further bolster the claim of viral persistence in wild bats, a population level study was performed to understand the circulation of zoonotic viruses in bat populations and the involved immune mechanisms (maternal antibody and acquired immune response) using mathematical modelling [45]. The study used sero-surveillance of an African henipavirus in straw-colored fruit bats (*Eidolon helvum*). While repeated introduction of virus and birthing of pups might drive viral dynamics in a large panmictic population of bats, prolonged infectious periods or latent infection of bats are required to explain henipavirus persistence in small populations (natural or colony of captive). They found that if repeated introduction of virus into small populations was the only mechanism, then acute infections would have to be ~40 days in duration. This estimate is considerably longer than the current estimates of the detectable infectious period for henipaviruses in fruit bats, which is approximately 7 days [46]. Therefore, prolonged or latent infection has to occur in some bats for describing persistence of henipavirus in small populations.

## 5. Stress-Induced Spillover—A Molecular Perspective

Spillover events are complex and usually require successful alignment of several contributing factors [47]. One such critical factor is increased shedding of virus by bats. This factor is influenced by the host response and virus replication. Plowright et al. [47] proposed a hypothesis which states that viruses infect naïve susceptible bats leading to acute infection. This subsequently progresses to a chronic or latent infection. The virus then reactivates from time to time in response to a variety of physiological and environmental triggers. This hypothesis is called the SILI hypothesis: Susceptible–Infectious–Latent–Infectious [47]. As described above, the bat immune system is unique and virus infection probably persists in many bats.

Several factors may alter long-term, low-level viral persistence in bats. Suppression of the immune response holding active virus replication in check would allow the virus to replicate to higher levels. This could happen in stressful conditions that affects the immune system. In other animals, a variety of stressors lead to reactivation of latent herpesviruses as reviewed by Grinde [48,49]. In neurons latently infected by herpes simplex virus -1 (HSV-1), viral replication is inhibited by the recruitment of CD8^+^ T cells which secrete interferon-γ (IFN-γ) and suppress viral transcription factors via noncytolytic granzyme mediated degradation [50]. During stress, there is a reduction of HSV-specific CD8^+^ T cells capable of producing IFN-γ. This contributes to reactivation of the virus [51]. Studies on murine gammaherpesvirus-68 (MHV-68) in mice have shown that latent gammaherpesviruses are sequestered in cells in the spleen and can be reactivated by stress [52,53]. Stressors, such as unfolded protein responses and hypoxia, can induce the expression of viral immediate early genes that help in initiating the lytic cycle of virus thereby reactivating it [54].

Arousal from hibernation is a stressful event for bats [55]. Many big brown bats are latently infected with a gammaherpesvirus [56]. Gerow et al. [57] demonstrated that the virus reactivates from latency when big brown bats arouse from hibernation, leading to detection of the virus in blood. This reactivation was also associated with a low level of antibodies against the virus. Following hibernation the antibody levels increase, which subsequently drives the virus into latency.

In pteropid bats, immunological stress was suggested as a contributing factor in henipavirus shedding [41]. As described earlier, Sohayati et al. [41] suspected that the recrudescence of NiV infection was triggered by waning antibody levels and an increased level of stress due to a combination of factors such as confinement in a cage, and physiological and behavioral changes during breeding season.

Secondary infections in humans are immunologically stressful [58]. Little brown bats are particularly susceptible to a frequently lethal fungal infection known as the white nose syndrome, caused by *Pseudogymnoascus destructans* [59,60,61]. A study looking at the effects of white-nose syndrome fungus on a persistently infecting coronavirus showed that bats having the fungal infection in wings had 60 times more coronavirus in their intestines as compared to fungal uninfected bats [62]. The intestines of the fungus-infected bats exhibited a gene expression profile suggesting suppression of the innate antiviral response, which may have contributed to unrestrained viral replication. This suggests that secondary infections in bats persistently infected with viruses could increase the potential of viral shedding.

**Figure 2:**
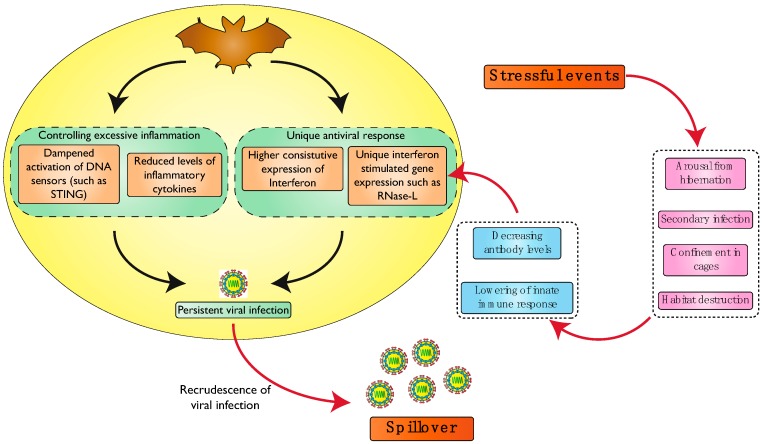
Model showing effect of stress on persistent viral infection. Viruses persistently infect bats due to their reduced inflammation (reduced DNA sensor activation and decreased inflammatory cytokine levels) and their effective antiviral immune response (increased constitutive expression of interferons and unique ISG expressions), as depicted in Figure 1. Stressful events alter the balance between host and virus and lead to an increase in virus replication, thereby leading to viral shedding.

These studies indicate that waning antibody levels and suppression of innate immune response due to stress might be some of the factors leading to an increase in viral levels in persistently infected bats (Figure 2).

## 6. Future Directions

The unique features of bat immune responses that promote viral persistence may exert evolutionary pressures on the virus as well. Bats have superseded rodents in harboring greater number of viruses and also having greater proportion of zoonotic viruses [63]. It is therefore crucial to understand how evolutionary pressure may have a role in the emergence of new viral strains. A recent study found that henipavirus genomes are best adapted to pteropid bats [64]. Adaptation of genomes refers to better capability of the virus to use host cellular machinery for its replication and protein synthesis, which is usually governed by natural selection; diversity in codon usage bias may contribute to it. Codon usage is an interspecies bias where one codon is selected over other synonymous codons in a particular species [65]. Natural selection for viral variants works by selecting codons matching host tRNA abundance. It also selects for variants with the advantage of not activating innate response genes, such as those for toll-like receptor 9. Codon bias analysis suggested that henipaviruses have the highest level of adaptation to pteropid bats. It would be interesting to study whether other viruses also show such codon bias towards their reservoir hosts. We might be able to use such codon bias studies in the future to identify reservoir hosts of spilled over viruses. Due to coevolution with the reservoir host, the viruses would have a codon bias specific towards their reservoir host. Apart from codon bias, natural selection based on receptor utilization also has a role to play in the evolution of viruses. Variation in the efficiency of bat coronaviruses to recognize human receptors show that the viral spike protein evolved in a stepwise manner to infect human cells [66]. Despite several other receptor-binding studies [67,68], the mechanism of adaptation to new hosts is not definitively understood.

Although there is some evidence for the increase in virus replication and shedding in bats under stress, a direct link of this to spillover events has yet to be discovered. Future controlled experiments aimed at studying transmission dynamics in the presence and absence of stress in bats would lead to a more definitive answer. It is also important to look into various factors that might stress bats such as habitat destruction (deforestation), pregnancy, change in seasons, and climate change. Additionally, the molecular mechanisms leading to the waning of antibodies and other aspects of adaptive immune response in bats are not known. A holistic picture of bat immune systems and the factors leading to an increase in viral replication might help us further understand viral spillovers.
